# *In vitro* antioxidative activity of yellow tea and its *in vivo* preventive effect on gastric injury

**DOI:** 10.3892/etm.2013.1117

**Published:** 2013-05-15

**Authors:** QIANG WANG, XIN ZHAO, YU QIAN, RUI WANG

**Affiliations:** Department of Biological and Chemical Engineering, Chongqing University of Education, Chongqing 400067, P.R. China

**Keywords:** yellow tea, antioxidant, Sprague-Dawley rat, cytokine, gastric injury

## Abstract

Yellow tea is a traditional Chinese drink widely used in Asia. The aim of this study was to determine the antioxidant activity of yellow tea and its preventive effect on gastric injury. The antioxidant effects were determined by measuring the 1,1-diphenyl-2-picryhydrazyl (DPPH) free radical- and hydroxyl radical-scavenging activity. The yellow tea extract demonstrated high antioxidant activity in the assays of DPPH and hydroxyl radical-scavenging activity. Additionally, an animal model was used to investigate the preventive effect of yellow tea on gastric injury. High concentrations of yellow tea reduced the levels of the serum pro-inflammatory cytokines interleukin (IL)-6 and tumor necrosis factor (TNF)-α to a greater extent than low concentrations. The extent of the gastric injury was significantly reduced by yellow tea, which demonstrated its anti-inflammatory properties. Yellow tea demonstrated the strongest inhibitory effect (74.6%) against gastric injury when administered at a dose of 1,000 mg/kg by gavage. These results suggest that yellow tea possesses good antioxidant activity and a preventive effect on gastric injury *in vivo*.

## Introduction

Yellow tea is a rare type of tea, which is slowly gaining recognition in Western countries. It is produced only in China and has a long history of use (1). Yellow tea usually implies a special tea processed similarly to green tea; however, it has a slower drying phase, where the damp tea leaves are allowed to sit and become yellow. This color is acquired by adding an extra step during production, called ‘sealed yellowing’, which is a slow oxidation process of tea polyphenols, including catechin (2). This unique step causes the tea to mellow and become bright yellow in color, without the grassy taste of green tea.

Antioxidants are substances that protect cells from damage caused by free radicals. Free radicals are molecules that have lost an electron and are therefore unstable (3). These free radicals take electrons from other molecules in order to stabilize themselves, ultimately creating new free radicals in the process. The removal of electrons may cause damage to DNA and lead to the possible development of inflammation (4).

Gastric injury is an injury to the stomach. It may be blunt or penetrating and may involve damage to the stomach organs. Ethanol promotes the rapid formation of injuries in the stomach, which occurs mainly due to an inflammatory reaction (5). Ethanol-induced gastric injury is characterized by epithelial cellular loss, mucosal edema and subepithelial hemorrhage (6). Cytokines, including interleukin (IL)-6 and tumor necrosis factor (TNF)-α, are small proteins that are produced and released from a number of cells under physiological and pathological conditions (7). IL-6 is increasingly recognized as an almost ubiquitous participant in numerous types of inflammatory processes (8). TNF-α is a macrophage-derived cytokine with chemotactic potency, which has been implicated in the acute phase reaction under various inflammatory conditions (9).

In the current study, the antioxidant activity of yellow tea and its preventive effect on gastric injury were examined. 1,1-Diphenyl-2-picryhydrazyl (DPPH) free radical- and hydroxyl (OH) radical-scavenging assays were conducted to evaluate the antioxidant activity of yellow tea. Additionally, measurements of the levels of the inflammation-related cytokines IL-6 and TNF-α were used to determine the preventive effects of yellow tea on HCl/ethanol-induced gastric injury in Sprague-Dawley rats.

## Materials and methods

### Preparations of yellow tea

Yellow tea was purchased from Sichuan Mengding Huangcha Tea Industry Co., Ltd., China. The yellow tea was stored at −80°C and freeze-dried to produce a powder. A twenty-fold volume of boiling water was added to the powdered sample and extraction was conducted twice. The aqueous extract was evaporated using a rotary evaporator (Eyela N-1100, Tokyo, Japan), concentrated and then dissolved in dimethylsulfoxide (DMSO; Amresco, Solon, OH, USA) to adjust to the stock concentration (20%, w/v).

### DPPH free radical assay

The DPPH free radical-scavenging activity was determined according to the method of Blois (10). Four milliliters of 100, 200 and 500 *μ*g/ml concentrations of the sample solution were added to 1.0 ml DPPH methanol solution (1.5×10^−4^ M). After storing at room temperature for 30 mins, the absorbance of the solution was determined at 520 nm using a spectrophotometer and the remaining DPPH was quantified. The results expressed are the means of triplicate values (11).

### OH radical assay

The OH radical-scavenging activity was assessed as described by Banerijee *et al* (12). The reaction system (1.4 ml), contained yellow tea extract, deoxyribose (6 mM, 0.2 ml), 0.2 ml sodium phosphate buffer solution (20 mM, pH 7.4), 0.2 ml anhydrous iron chloride (FeCl_3_; 400 *μ*M), 0.2 ml FeSO_4_-ethylenediaminetetraacetic acid (EDTA; 400 *μ*M), 0.2 ml H_2_O_2_ (3 mM), 0.2 ml ascorbic acid (400 *μ*M) and 0.2 ml yellow tea extract solution (50, 100 and 200 *μ*g/ml). After incubation at 37°C in a water bath for 60 min, the reaction was stopped by adding 1 ml trichloroacetic acid and 1 ml 2-thiobarbituric acid in a 1.4-ml reaction system. The solution was boiled for 20–25 min at 90°C in a water bath. The absorbance was measured at 532 nm. All analyses were run in triplicate and averaged.

### Animals

Male Sprague-Dawley rats (n=50, 7-weeks-old) were purchased from the Experimental Animal Center of Chongqing Medical University (Chongqing, China). The rats were maintained in a temperature-controlled facility (temperature, 25±2°C; relative humidity, 50±5%) with a 12-h light/dark cycle and free access to a standard rat chow diet and water.

### Gastric injury assay

The experimental design was as follows: the normal and control groups received 14-day repeated oral administration of distilled water and a single dose of the vehicle (2 ml/kg b.w. olive oil, p.o.); the sample groups received 14-day repeated oral administration of 250, 500 or 1,000 mg/kg yellow tea extract. Then, the control and sample group rats were administered 1 ml HCl/ethanol (60% in 150 mM HCl) p.o. through esophageal intubation and were then sacrificed 1 h later under deep ether anesthesia. The stomachs were removed, inflated by injecting 10 ml 1% formalin for 10 min to fix the tissue walls and opened along the greater curvature. The area (mm^2^) of hemorrhagic lesions developed in the stomach was measured using a digital camera (D550; Canon, Tokyo, Japan) with a square grid and the images were analyzed by ImageJ software (National Institutes of Health, Bethesda, MD, USA). These experiments followed a protocol approved by the Animal Ethics Committee of Chongqing Medical University (Chongqing, China).

### Analysis of inflammation-related cytokines in serum by enzyme-linked immunosorbent assay (ELISA)

For the serum cytokine assay, blood from the inferior vena cava was collected in a tube and centrifuged (1,370 × g for 10 min at 4°C). The serum was aspirated and assayed as follows: Concentrations of the inflammatory-related cytokines IL-6 and TNF-α in serum were measured by ELISA according to the kit manufacturer’s instructions (BioLegend, San Diego, CA, USA). Briefly, after the biotinylated antibody reagent was added to 96-well plates, supernatants of homogenized serum were incubated at 37°C in CO_2_ for 2 h. After washing with phosphate-buffered saline (PBS), horseradish peroxidase (HRP)-conjugated streptavidin peroxidase solution was added and the plate was incubated for 30 min at room temperature. The absorbance was then measured at 450 nm using a microplate reader (13).

### Statistical analysis

Data are presented as mean ± standard deviation (SD). Differences between the mean values for individual groups were assessed with one-way analysis of variance (ANOVA) with Duncan’s multiple range test. P<0.05 was considered to indicate a statistically significant difference. SAS version 9.1 (SAS Institute Inc., Cary, NC, USA) was used for statistical analyses.

## Results

### DPPH radical-scavenging activity

**Figure 1.**
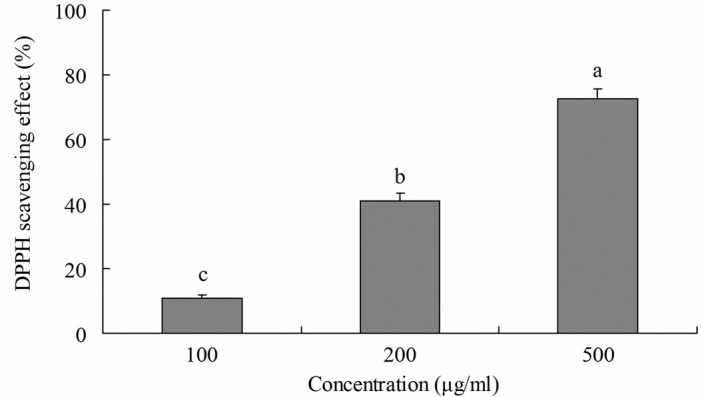
1,1-Diphenyl-2-picryhydrazyl (DPPH) free radical-scavenging activity of yellow tea. ^a,b,c^Mean values with different letters over the bars are significantly different (P<0.05) according to Duncan’s multiple range test.

The radical-scavenging activity of yellow tea was investigated using DPPH radicals (Fig. 1). Yellow tea showed scavenging activity on DPPH radicals at various concentrations. At 100, 200 and 500 *μ*g/ml, the radical-scavenging activities were 10.8, 41.0 and 72.6%, respectively. This indicates that the radical-scavenging activity of yellow tea increased as the concentration of its extract increased.

### OH radical-scavenging activity

**Figure 2.**
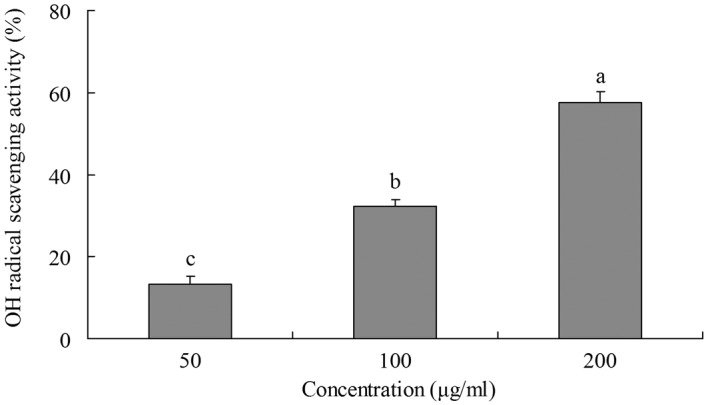
Hydroxyl radical-scavenging activity of yellow tea. ^a,b,c^Mean values with different letters over the bars are significantly different (P<0.05) according to Duncan’s multiple range test.

The ability of yellow tea to scavange OH radicals was evaluated by measuring the deoxyribose damage induced by the Fe^3+^/ascorbate/EDTA/H_2_O_2_ system using the thiobarbituric acid (TBA) method (14). Deoxyribose degrades into fragments that react with TBA upon heating at a low pH to form a pink color. The inhibitory effects of yellow tea on deoxyribose damage are shown in Fig. 2. The inhibitory rate of a 200 *μ*g/ml extract was 57.6%, which is higher than those of 100 *μ*g/ml (32.2%) and 50 *μ*g/ml (13.2%) extracts.

### Inflammation-related cytokine levels in serum

**Figure 3.**
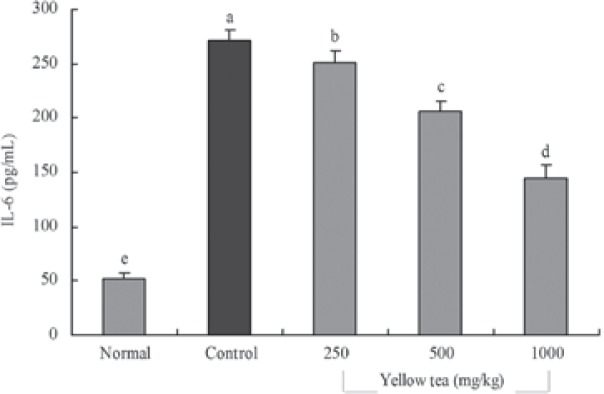
Effect of yellow tea on the serum interleukin (IL)-6 level in rats with HCl/ethanol-induced gastric injury. ^a–e^Mean values with different letters over the bars are significantly different (P<0.05) according to Duncan’s multiple range test.

**Figure 4.**
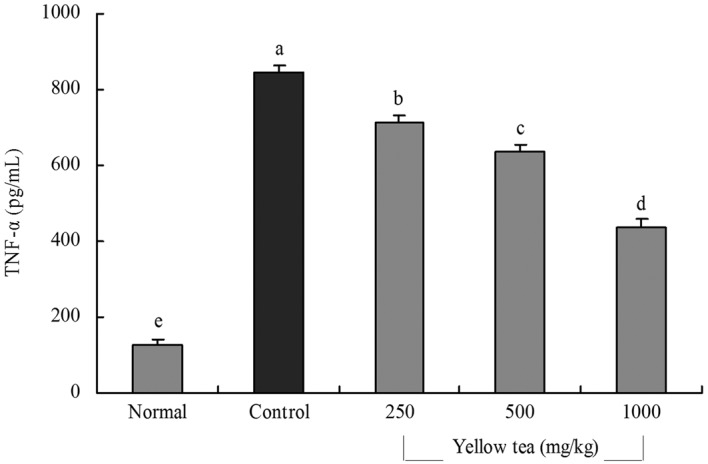
Effect of yellow tea on the serum tumor necrosis factor (TNF)-α level in rats with HCl/ethanol-induced gastric injury. ^a–e^Mean values with different letters over the bars are significantly different (P<0.05) according to Duncan’s multiple range test.

The IL-6 level of normal rats was 52.1±4.5 pg/ml; however, that of control rats was significantly increased to 271.2±10.2 pg/ml following the induction of gastric injury. The levels of IL-6 in rats fed with 250, 500 and 1,000 mg/kg yellow tea were 250.7±11.2, 206.3±9.8 and 144.3±12.2 pg/ml, respectively (Fig. 3). The TNF-α levels in normal, control and 250, 500 and 1,000 mg/kg yellow tea-treated rats were 127.5±14.6, 843.3±22.2, 715.3±18.5, 635.4±20.6 and 438.4±19.4 pg/ml, respectively (Fig. 4). The serum IL-6 and TNF-α levels in the rats in the yellow tea-treated groups were significantly lower than those in the control group.

### Gastric injury levels

**Figure 5.**
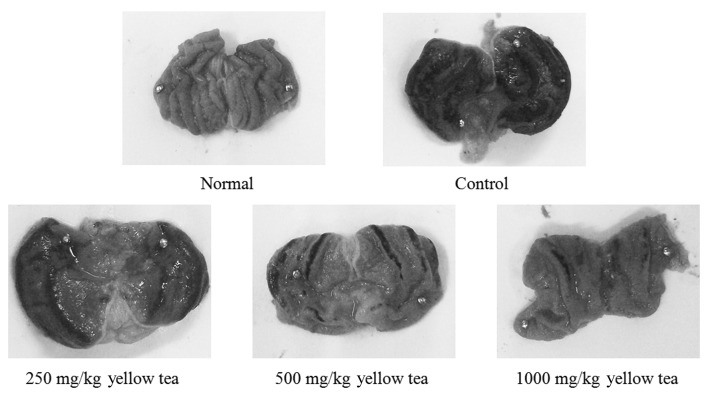
Appearance of the stomach following HCl/ethanol-induced gastric injury in rats treated with various concentrations of yellow tea.

The administration of yellow tea to rats prior to the induction of gastritis led to reduced gastric injury. The rats of the control group demonstrated a gastric injury area of 14.2±3.4 mm^2^. Treatment with 250 and 500 mg/kg yellow tea resulted in gastric injury inhibition rates of 11.3% (gastric injury area, 12.6±2.2 mm^2^) and 56.3% (gastric injury area, 6.2±1.6 mm^2^), respectively, while 1,000 mg/kg yellow tea (inhibition rate, 74.6%; gastric injury area, 3.6±1.1 mm^2^) demonstrated the best gastritis preventive effect (Table I and Fig. 5). The results suggest that yellow tea has a strong preventive effect on gastric injury.

## Discussion

Although yellow tea is a traditional drink, little scientific data on its effects are available. Yellow tea is a type of fermented tea. Since a large number of digestive enzymes are generated during its smothering process, a slow oxidation process, yellow tea is beneficial for the spleen and stomach. A previous study demonstrated that yellow tea, which is rich in tea polyphenols, polysaccharides, vitamins and amino acids, has special effects for preventing and curing esophageal cancer (2).

The DPPH assay is based on the capacity of a substance to scavenge stable radicals. It has been widely used to test the ability of compounds or plant extracts to act as free radical-scavengers or hydrogen donors (11). DPPH is a stable free radical (purple in color) which may accept an electron or hydrogen radical to become a stable yellow diamagnetic molecule. It is widely used to predict the potential antioxidative capability of foods and plant extracts *in vitro*. The highly reactive OH radical causes oxidative damage to DNA, proteins and lipids, which contribute to inflammation, mutagenesis and cytotoxicity (15).

The serum levels of cytokines, including IL-6, TNF-α, IL-1β and interferon (IFN)-γ, in patients with inflammatory diseases are higher compared with those in healthy individuals (16). Thus, lower levels of IL-6 and TNF-α are indicative of anti-inflammatory effects and yellow tea demonstrates a good protective effect against gastric damage. Hepatocytes bear a variety of cytokine receptors. IL-6 is an interleukin that acts as a pro-inflammatory and anti-inflammatory cytokine. In humans, it is encoded by the IL-6 gene (17). IL-6 is secreted by T cells and macrophages to stimulate the immune response, particularly in the process of tissue damage leading to inflammation. IL-6 also plays a role in fighting infection (18). TNF-α is a cytokine involved in systemic inflammation and is a member of a group of cytokines that stimulate the acute phase reaction. The primary role of TNF-α is in the regulation of immune cells. TNF, as an endogenous pyrogen, is able to induce fever, induce apoptotic cell death, sepsis (through IL-1 and IL-6 production), cachexia and inflammation, as well as inhibit tumorigenesis and viral replication (19). The inflammatory cytokines IL-6 and TNF-α play pathogenic roles in diseases of the stomach (20). Although systemic IL-6 levels are elevated following traumatic hemorrhage, hepatocellular function is impaired and gastric injury occurs (21). TNF-α is also a key mediator in a number of experimental models of stomach injury (22).

The current study demonstrates that yellow tea has antioxidant activity and is effective in the prevention of HCl/ethanol-induced gastric injury in Spraque-Dawley rats. Our results demonstrate that the protective effects of yellow tea may be due to its antioxidant activity and reductions in the levels of pro-inflammatory cytokines, including IL-6 and TNF-α. The appearance of the stomach also indicated that yellow tea is able to prevent HCl/ethanol-induced gastric injury. These results suggest that yellow tea has *in vitro* anti-oxidant effects and is potentially useful in the treatment or prevention of chemical-induced gastric injury *in vivo*.

## Figures and Tables

**Table I.** Preventive effect of yellow tea treatment against HCl/ethanol-induced gastric injury.

	Gastric injury data	
Group	Gastric injury (mm^2^)	Inhibition rate (%)
Normal	0.0±0.0^c^	100.0
Control	14.2±3.4^a^	0.0
Yellow tea (mg/kg)		
250	12.6±2.2^c^	11.3
500	6.2±1.6^c^	56.3
1000	3.6±1.1^c^	74.6

a–c Mean values with different letters in the same column are significantly different (P<0.05) according to Duncan’s multiple range test.
